# Application of mid-infrared (MIR) microscopy imaging for discrimination between follicular hyperplasia and follicular lymphoma in transgenic mice

**DOI:** 10.1039/c5an01072a

**Published:** 2015-09-21

**Authors:** C. Woess, M. Drach, A. Villunger, R. Tappert, R. Stalder, J. D. Pallua

**Affiliations:** a Institute of Legal Medicine, Medical University of Innsbruck, Müllerstraße 44, 6020 Innsbruck, Austria; b Institute of Dermatology, University Hospital, Zurich, Switzerland; c Institute of Pathology, University Hospital, Zurich, Switzerland; d Division of Developmental Immunology, BIOCENTER, Medical University Innsbruck, Austria; e Institute of Mineralogy and Petrography, Leopold-Franzens University Innsbruck, Innrain 52f, 6020 Innsbruck, Austria

## Abstract

Mid-infrared (MIR) microscopy imaging is a vibrational spectroscopic technique that uses infrared radiation to image molecules of interest in thin tissue sections. A major advantage of this technology is the acquisition of local molecular expression profiles, while maintaining the topographic integrity of the tissue. Therefore, this technology has become an essential tool for the detection and characterization of the molecular components of many biological processes. Using this method, it is possible to investigate the spatial distribution of proteins and small molecules within biological systems by *in situ* analysis. In this study, we have evaluated the potential of mid-infrared microscopy imaging to study biochemical changes which distinguish between reactive lymphadenopathy and cancer in genetically modified mice with different phenotypes. We were able to demonstrate that MIR microscopy imaging and multivariate image analyses of different mouse genotypes correlated well with the morphological tissue features derived from HE staining. Using principal component analyses, we were also able to distinguish spectral clusters from different phenotype samples, particularly from reactive lymphadenopathy (follicular hyperplasia) and cancer (follicular lymphoma).

## 1. Introduction

Follicular lymphoma is one of the most common subtypes of indolent lymphoma. Therefore, most patients are diagnosed in an advanced stage and there is still no standard therapy fitting all patients.^1^ The genetic hallmark of this disease is a t(14;18) translocation and the subsequent overexpression of the anti-apoptotic protein B-cell lymphoma 2 (Bcl2), leading to a survival advantage of B-cells and, therefore, playing a crucial role in the pathogenesis of follicular lymphoma.^2,3^ This translocation is observed in 75–90% of follicular lymphoma patients.^2,4^ It is the result of changes in the genetic program that can lead to developmental arrest and/or uncontrolled proliferation of B-cells in a certain stage of progress. These clones are from the germinal centre type, thus centrocytes and centroblasts.^4^ Follicular hyperplasia, on the other hand represents the most common type of reactive lymphadenopathy. Although there are some indicators that can help differentiate between cancer (follicular lymphoma) and hyperplasia, such as the patient’s history and age, or even some histological characteristics (*e.g.* density of follicles), there is no pathognomonic histologic feature.^5^

To gain a deeper insight into the distinction between follicular hyperplasia and follicular lymphoma, we analyzed tissues from Vav-Bcl2 transgenic mice. These mice overexpress Bcl2 in the hematopoietic system, leading to a predisposition to suffer from follicular lymphoma and subsequently premature death.^2^ It has been shown that the overexpression of a transmembrane activator and a CAML interactor receptor, coupled to the Fc-fragment of human immunoglobulin G (TACI-Ig) (causing the neutralization of the survival and maturation factor for B-cells, BAFF (B-cell activating factor of the TNF family)) is able to decrease the number of B-cells in Vav-Bcl2 mice, which alleviates disease burden and subsequently prolongs the survival of these mice. Histological evaluation of spleens from double-transgenic mice indicated reduced follicle and germinal center size.^6^

To distinguish follicular lymphoma (in Vav-Bcl2 mice) from follicular hyperplasia (in Vav-Bcl2/TACI-Ig mice), we used a bioanalytical technique for its investigation, in particular mid-infrared (MIR) imaging.^7-10^

This modern imaging method is regarded as a promising analytical tool for environmental mapping, product functionality,^11,12^ determining the severity of plant diseases,^13,14^ detecting defects^12^ and contamination,^15,16^ as well as determining the distribution of certain chemical components.^17-23^ Emerging biomedical applications in MIR imaging also include tissue histopathology, which has been proposed as a solution for histopathological differentiation between normal, benign and malignant tissue.^24,25^ This imaging method has already demonstrated great promise for the detection and characterization of malignancies in several tissues, including skin,^26^ cervix,^27^ esophagus,^28^ stomach,^29^ lung,^30^ ovary,^31^ prostate,^32-34^ colon,^35^ brain^36-39^ and squamous epithelia.^40^ It has been applied to study individual cells at a subcellular spatial resolution and allows determination of the state of chemical bonding and mapping of the relative concentration of the lipid, protein, carbohydrate and phosphorylated molecular domains, across the cell.^41-43^ A major advantage of this technique is the acquisition of local molecular expression profiles, while maintaining the topographic integrity of the tissue and avoiding time-consuming extraction, purification, and separation steps.^34,44^ The greatest benefit lies in the high molecular sensitivity combined with a spatial resolution down to a few micrometers. Hence, MIR imaging is a powerful tool in histopathological characterization. It allows the investigation of the spatial distribution of proteins and small molecules within biological systems by *in situ* analysis of tissue sections without the use of staining and with minimal sample preparation effort.^8,34,45-49^ Monitoring capabilities of this method are based on the vibrational excitation of chemical bonds by IR radiation detecting biochemical changes during tumor development (*i.e.*, more DNA, less protein, or changes in carbohydrates).^24,25^

The resulting spectral features (amide I region, amide II region, lipid regions, carbohydrates, DNA/RNA and α-helical structures) can provide a characteristic spectrum of infrared absorption peaks, representing a molecular fingerprint of the biochemical composition of the tissue.^50-52^

In order to gain more insight into MIR follicular lymphoma pathology, a detailed investigation into the spectral cytology of mouse spleens from various genotypes was deemed necessary. The investigation aimed at understanding the spectral characteristics of abnormalities by observing the origin of major spectral types in follicular lymphoma. MIR microscopy imaging, in conjunction with multivariate data analysis, was applied to the analysis of spleen tissue in an attempt to distinguish between cancer and reactive hyperplasia. The main goal of this study was to identify spectral characteristics through correlating different spleen phenotypes (normal tissue, follicular hypoplasia, follicular hyperplasia and follicular lymphoma) that can be used to predict a region-specific susceptibility to follicular lymphoma.

## 2. Material and methods

### 2.1 Materials

Octane (≥99.0%) from Sigma Aldrich (St. Louis, MO, USA), hematoxylin (hematoxylin solution according to Mayer) from Sigma Aldrich (St. Louis, MO, USA) and eosin (Eosin Y) from Sigma Aldrich (St. Louis, MO, USA) were used for the sample preparation. All samples were mounted on infrared-transparent CaF2 slides, 1 mm thick (KORTH KRISTALLE GmbH, Altenholz, Germany) and 1 mm thick (Menzel slides, Fisher Scientific, Vienna, Austria).

### 2.2 Mouse strains and tissue preparation

C57BL/6 TACI-Ig transgenic and Vav-Bcl2 transgenic mice have been described elsewhere.^53,54^ Organs were fixed in 4% PFA (paraformaldehyde) in phosphate-buffered saline (PBS), processed according to standard procedures.

### 2.3 Assessment of spleen sections

HE-stained slides from the various mouse genotypes (C57BL/6, TACI-Ig transgenic, Vav-Bcl2 and Vav-Bcl2/TACI-Ig) were evaluated by a pathologist.

### 2.4 MIR microscopy imaging

Mouse spleens from four different genotypes (wt, TACI-Ig, Vav-Bcl2 and Vav-Bcl2/TACI-Ig) were chosen for the MIR microscopy imaging study. For preparing tissue sections, the blocks were fixed on a microtome and two tissue sections of 4 μm thickness were cut; one was stained with hematoxylin and eosin (HE) for histological validation by a pathologist and the other one was used for the MIR microscopy imaging study. The tissue sections for the MIR microscopy imaging study were de-paraffinized with octane at 40 °C in a water bath by moderate shaking for 4 h.^23^ Thereafter, the slides were dried in an aspirator (3.2 kPa) for 30 min at room temperature and measured with a MIR microscope. The drying time proved to be sufficient as prolonging the drying time to 24 h caused no difference in spectra quality.^23^ Spectroscopic imaging data of the tissue sections were acquired at room temperature in transmission mode using a Bruker Vertex 70 Fourier transform infrared (FTIR) spectrometer, coupled to a Hyperion 3000 microscope, which was equipped with a usual nitrogencooled MCT-D316-025 (mercury cadmium telluride) detector called a single-element detector and a nitrogen-cooled focal plane array (FPA) detector consisting of 64 × 64 MCT-D364 detectors. The spectrometer was continuously flushed with dried air to minimize the water-vapor background. Visual image collection was performed *via* a video camera integrated in the microscope stage. Spectral data were recorded using the FPA detector with nominal lateral pixel resolution of 2.65 μm × 2.65 μm and a spectral resolution of 4 cm^−1^ with 32 co-added scans. The detector range was set from 3900 cm^−1^ to 850 cm^−1^. Before each sample measurement, an appropriate background spectrum was collected outside the sample area. After measurement, the sections were stained with HE for histological reevaluation and compared with the imaging results by the pathologist.

### 2.5 Data processing

All spectral data processing and image assembling were performed using The Unscrambler X 10.2 (Camo, Norway, Oslo) and the CytoSpec^™^ software package (http://www.cytospec.com, Hamburg, Germany).

#### 2.5.1 Principal component analyses (PCA)

Before principal component analyses (PCA) and image analysis, it was necessary to remove atmospheric absorptions and noise by using the CytoSpec^™^ software package (http://www.cytospec.com, Hamburg, Germany).

PCA models were generated with The Unscrambler X 10.2 software, after atmospheric correction and noise reduction. For PCA model generation, tissue type-associated spectra were selected using the CytoSpec^™^ software. For this purpose, we evaluated the sample and defined the regions of interest (ROIs). The extracted spectra of ROIs were imported into The Unscrambler X 10.2 software and underwent several data pre-treatments (*e.g.*, baseline correction, normalization), before PCA model generation.

#### 2.5.2 Mid-infrared (MIR) microscopy image analysis

For univariate and multivariate image analyses (MIAs), MIR images were loaded into the CytoSpec^™^ software. Before image analyses, it was necessary to remove atmospheric absorptions and noise by using the CytoSpec^™^ software. After this pre-treatment, spectra were vector-normalized and smoothed (Savitzky–Golay, 13 smoothing points) in the wave number range from 3900 cm^−1^ to 850 cm^−1^. These procedures led to better resolved peaks, eliminated background slopes, and reduced the influence of intensity changes caused by the differences in tissue density and roughness of the tissue.^4^

Univariate image analyses, depicting a single spectral feature of the data set, were used to reproduce the actual morphology. This strategy provides only a partial representation of the obtained imaging data with a minimal computation effort.^55,56^ Single spectral features displaying essential differences (1740 cm^−1^, 1155 cm^−1^ and 1080 cm^−1^) were used for comparison among the spleen samples.

For further image analyses, MIAs were performed to fully characterize the range of spectral variations.^55,57-59^ MIAs, such as hierarchical clustering (HCA), K-means (KMC) clustering and fuzzy C-means clustering (FCM) in the spectral ranges 3650 cm^−1^ to 3050 cm^−1^, 3000 cm^−1^ to 2800 cm^−1^ and 1750 cm^−1^ to 850 cm^−1^ were used for data analyses. Furthermore, the results of univariate analyses and of MIAs were assembled and compared directly with the HE images taken from the same samples. Different clustering techniques were used to find the best method, which is able to reproduce the actual morphology. For detailed information about univariate image analysis, as well as MIAs theory and current developments, the interested reader is referred to the cited literature.^24-33,44,60-67^

## 3 Results and discussion

**Fig. 1:**
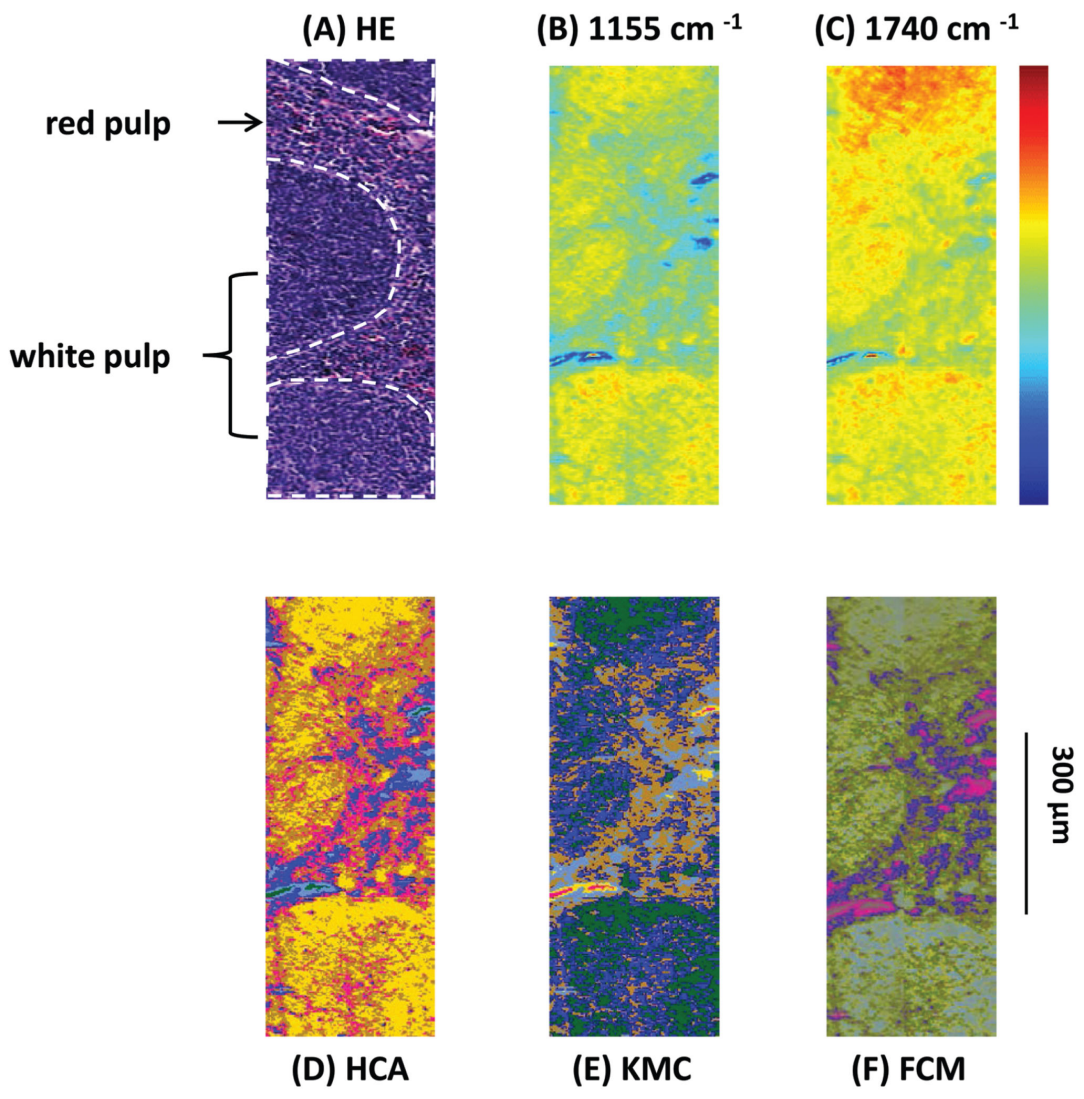
(A) Light microscopy image of a labelled HE-stained tissue section of a sample from a Vav-Bcl2/TACI-Ig mouse spleen. White pulp (white indication), consisting of lymphoid follicles with B-lymphocytes and follicular dendritic cells and the red pulp, which is rich in blood and vessels, can be differentiated. (B) MIR imaging result shown in false colour representation. Colours reflect intensities of the selected absorption at 1155 cm^−1^, which is commonly attributed to carbohydrates. (C) MIR imaging result shown in false colour representation. Colours reflect intensities of the selected absorption at 1740 cm^−1^, which is commonly attributed to *ν*_C=O_ esters, phospholipids as well as carbohydrates. (D) Hierarchical cluster analysis. (E) K-means clustering image. (F) Spectroscopic image of the fuzzy C-means clustering.

**Fig. 2:**
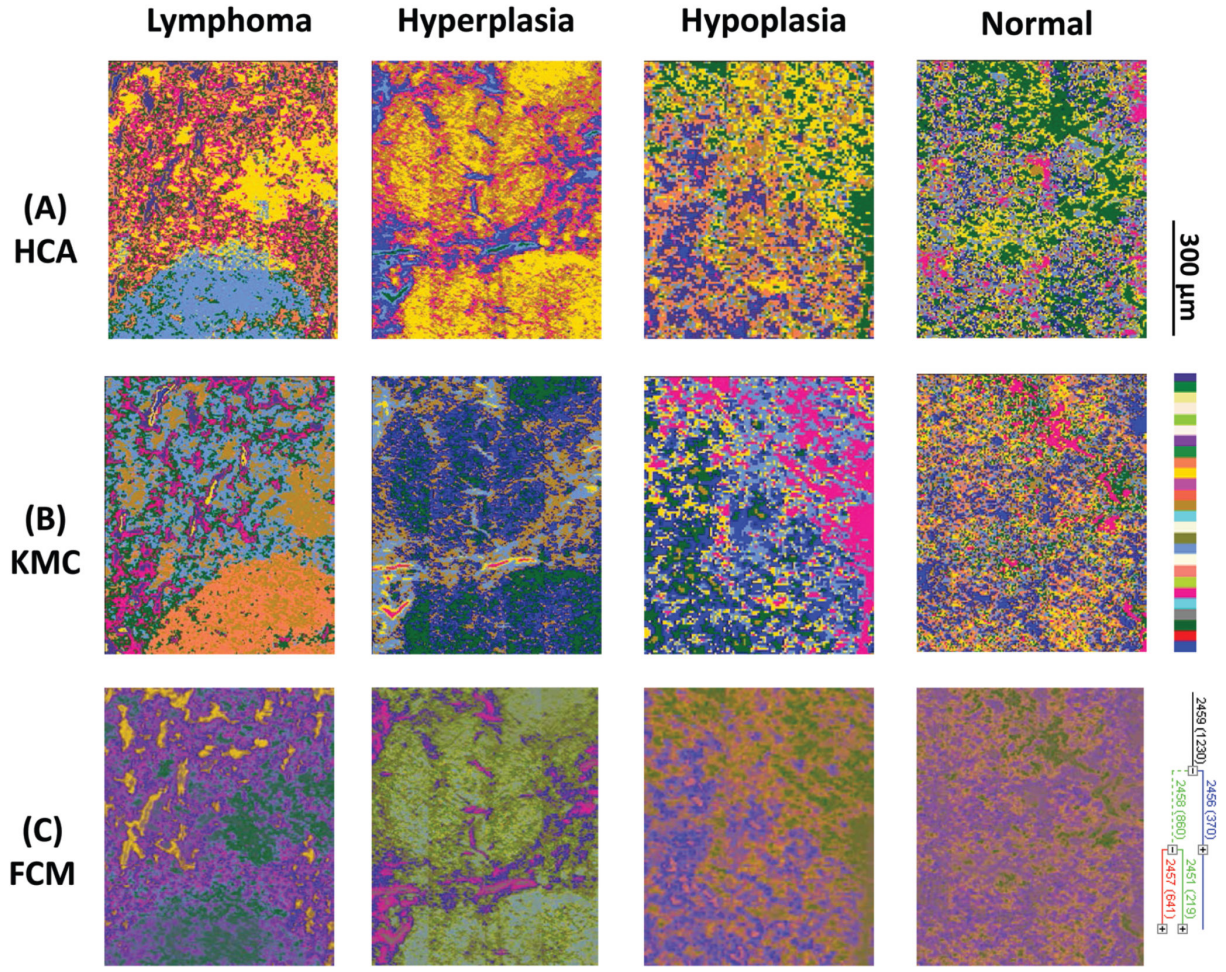
(A)–(C) Infrared spectroscopic chemical-maps obtained for the detection of *ν*_P=O_ symmetric vibrations at 1080 cm^−1^, of carbohydrates at 1155 cm^−1^ and of *ν*_C=O_ esters, phospholipids as well as carbohydrates at 1740 cm^−1^. The spleen samples belong to the following mouse genotypes: lymphoma (Vav-Bcl2), follicular hyperplasia (Vav-Bcl2/TACI-Ig), follicular hypoplasia (TACI-Ig) and normal (wt).

**Fig. 3:**
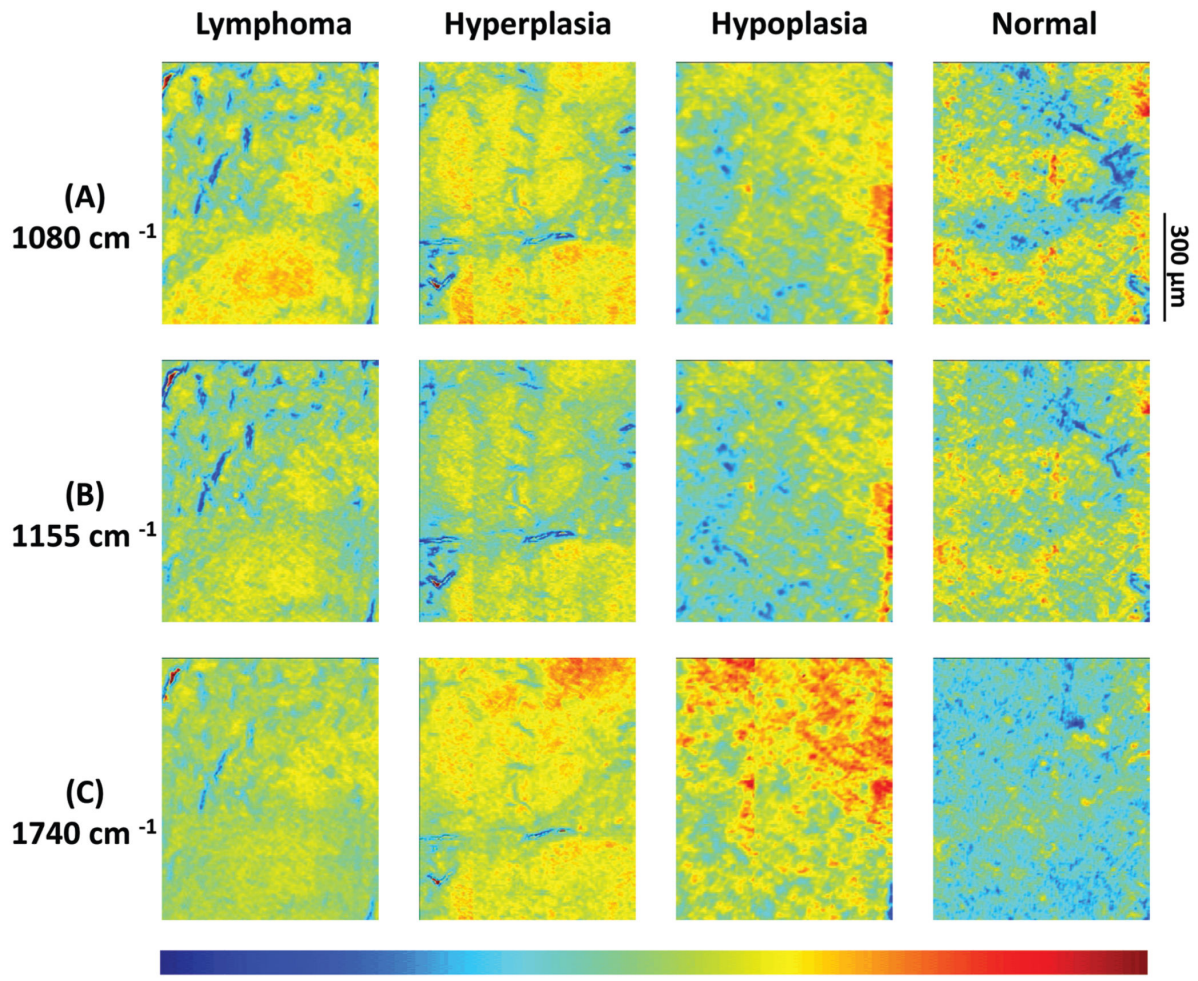
(A) Hierarchical cluster analyses, (B) K-means clustering images and (C) fuzzy C-means clustering of four individual mouse genotypes with different phenotypes: lymphoma (Vav-Bcl2), follicular hyperplasia (Vav-Bcl2/TACI-Ig), follicular hypoplasia (TACI-Ig) and normal (wt).

**Fig. 4:**
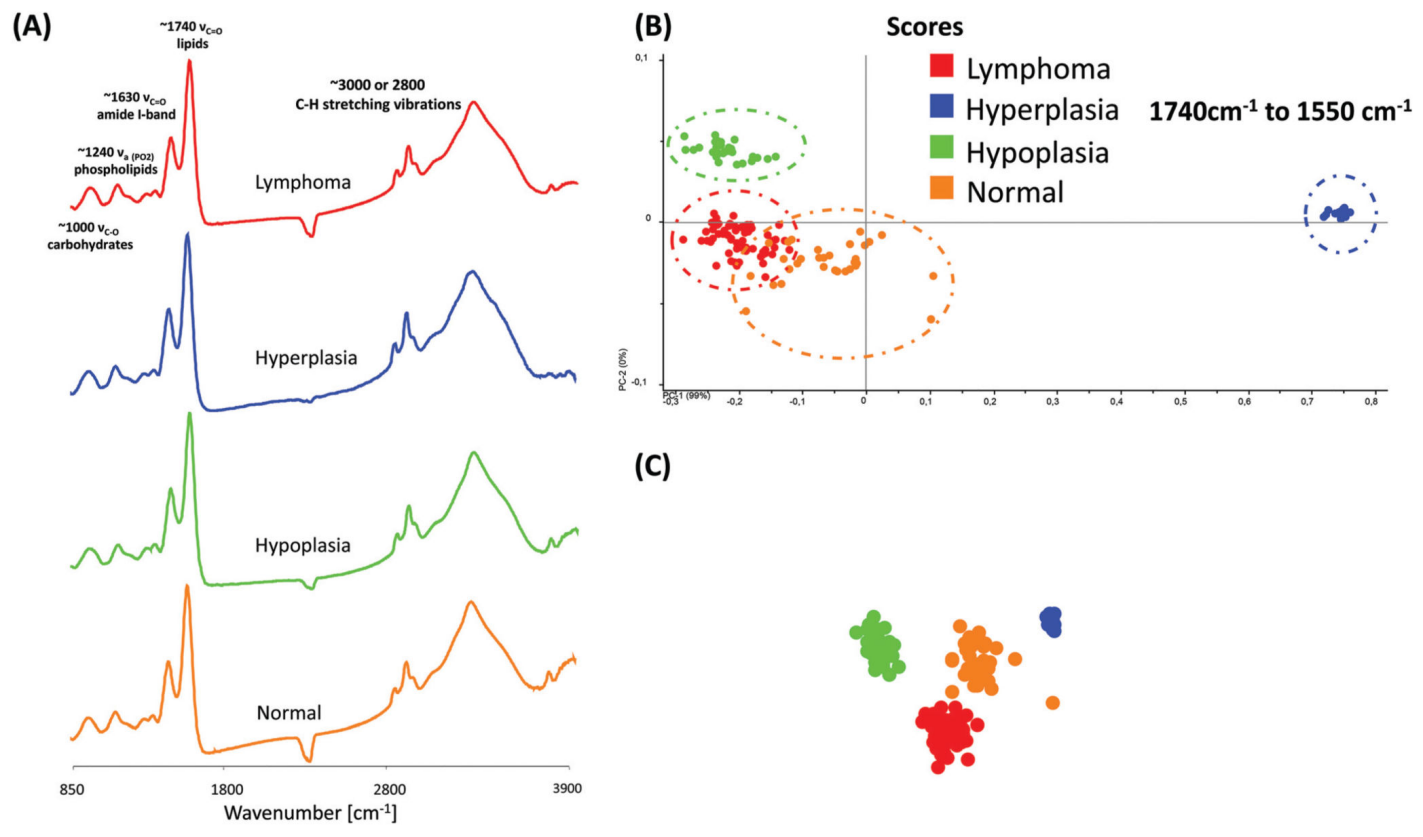
(A) Representative MIR spectra of different phenotype samples. (B)–(C). 2-D and 3-D score plot of MIR spectra in the region from 1740 cm^−1^ to 1550 cm^−1^. For the differentiation between the different phenotypes by PCA, 30 spectra were selected from 4 individual phenotype samples. Each data point represents one spectrum of the respective (colour coded) spleen sample.

Analyses of the resulting MIR microscopy imaging data sets were performed using the before mentioned software packages. In this study, tissue samples were analysed by spectra-analysis, individual principal component analyses (PCA), and individual multivariate image analyses (MIAs). Results of single tissue samples are depicted in Fig. 1-4.

Results presented in Fig. 1 illustrate the capability of spectroscopic imaging to accurately reproduce tissue histology of the Vav-Bcl2/TACI-Ig mouse spleen.

In Fig. 1(A)–(F) comparison of the measured HE-stained tissue sections, generated chemical maps and MIAs from one particular mouse tissue sample is illustrated. The image displayed in Fig. 1(A) was collected from a tissue section measured by MIR imaging with a nominal lateral resolution of 2.65 μm × 2.65 μm per pixel for each spot (chemically dewaxed with octane for 4 h) and stained afterwards with HE. The image of the HE-stained slide was then directly compared with the images constructed from chemical maps (Fig. 1(B) and (C)) or cluster analyses (Fig. 1(D)–(F)). Different tissue types can be recognized in Fig. 1(A): the white pulp, which primarily consists of lymphoid follicles (mainly composed of B-lymphocytes and follicular dendritic cells), represents a very active area. There, B-lymphocytes get activated by antigens and subsequently differentiate into centroblasts (still able to divide) and proliferate, for this reason the zone is very dark. During this expansion, somatic hypermutation (SHM) modifies the affinity of the B-cell receptor. If the result is unwanted, the cell is eliminated by apoptosis (programmed cell death). If the result improves the affinity of the B-cell receptor, centroblasts differentiate into centrocytes, and move to the light zone, where, with the help of T-cells and follicular dendritic cells (FDCs), they are selected and become memory B-cells or plasma cells.^68^ Furthermore, there is the red pulp (abundant with blood and vessels), which consists of reticular connective tissue with fibroblastic reticular cells and reticular fibres. In these pictures, the histological appearance of follicular hyperplasia (from Vav-Bcl2/TACI-Ig mice) is shown. Fig. 1(B) depicts a chemical map generated by integrating the area under the absorption band at 1155 cm^−1^, which is commonly attributed to carbohydrates. The result correlates well with the morphology of the lymphoid follicles and the surrounding red pulp, indicating that these tissue types produce high amounts of carbohydrates, representing a very active region. The chemical map of the absorption at 1740 cm^−1^ is attributed to *ν*_C=O_ esters, phospholipids as well as carbohydrates (Fig. 1(C)). These observations indicate that the white pulp *i.e.*, lymphoid follicles, is highly metabolic with a high proliferation rate, compared to the surrounding red pulp. Specific correlations with morphological and histological features, however, cannot be produced with this form of processing, because biological structures are composed of a variety of complexly constructed macromolecules. The spectral contributions mainly derive from three groups of substances: proteins, nucleic acids and lipids. The protein spectra in the mid-infrared range have different characteristic bands, whose vibrations can be assigned to the amino acid side groups^69^ or to the peptide backbone.^70^ The spectroscopic absorption bands of lipids are the C–H stretching and deformation vibrations of the >CH_2_ and −CH_3_ groups, and the ester carbonyl bands and the PO_2_^−^ bands of biological membranes. MIR spectra of nucleic acids are often classified into four spectral ranges: (a) 1780 cm^−1^ to 1550 cm^−1^ in-plane vibrations of double bonds of the bases, (b) 1550 cm^−1^ to 1270 cm^−1^ deformation vibrations of the bases which are coupled with the sugar vibrations, (c) 1270 cm^−1^ to 1000 cm^−1^ two strong absorption bands of PO_2_^−^ (asymmetric and symmetric) and (d) 1000 cm^−1^ to 780 cm^−1^ vibrations of the sugar–phosphate backbone. Therefore, the IR spectra of biological samples are characterized by extreme superposition of many different IR bands. This fact makes it difficult to directly interpret IR spectra in many cases. Only in individual cases (*e.g.*, collagen of the connective tissue, and storage compounds of microorganisms) direct conclusions on the basis of specific bands from the spectra can be drawn. Therefore, individual band parameters such as discrete frequency or extinctions are, in most cases, of limited use to characterize and to identify the biological material. Nevertheless, with MIR spectrometry unique information on the molecular structure, mainly for determining the secondary structure of proteins,^71^ mutations of nucleic acids^72^ and peroxidation of phospholipids^73^ can be demonstrated. To fully decode the heterogeneous samples, multivariate statistical methods for data evaluation are used, which can discriminate between healthy *versus* pathological samples.^74,75^ Therefore, MIR data can be used as molecular signatures for determining the physiological status once the spectral patterns are correlated with biological properties.^76^ Consequently, different cluster analyses were performed to fully characterize the range of spectral variations through the tissue section.

Fig. 1(D) depicts a pseudo-colour image that was constructed by using hierarchical cluster analysis (HCA). The displayed image represents a six-cluster structure, reproducing histological features of the measured tissue section. A K-means clustering (KMC) image is presented in Fig. 1(E). The principal correspondence between the histological image and the KMC image is obvious; most of the spectral clusters can be assigned to the histological structures mentioned above. In Fig. 1(F) the result of fuzzy C-means (FCM) clustering is illustrated. Most of the individual colour in this spectroscopic image can be assigned to specific tissue structures, similar to the result of the KMC. Clustering analysis allows identification of tissue structures, but it should be noted, that an additional discrimination between histological structures has not been possible so far. Even if the number of clusters were increased (data not shown), further differentiation between histological structures could not be achieved.

Results from 4 individual genotype samples by chemical-maps and MIAs are illustrated in Fig. 2 and 3. The output of the data analyses illustrates the ability of spectroscopic imaging to reflect the differences of various phenotypes, especially between cancer (follicular lymphoma) and reactive lymphadenopathy (follicular hyperplasia) with a nominal lateral resolution of 2.65 μm × 2.65 μm per pixel for each spot. The depicted phenotypes of various genotypes are presented. Starting from the right, there is normal splenic architecture from wt (C57BL/6) mice, with the above-mentioned white and red pulp. The picture to the left (hypoplasia) illustrates diminished follicles, as a result of the smaller number of B-lymphocytes, due to the TACI-Ig expression, leading to B-cell loss, as published in ref. 53. Hyperplasia means that Vav-Bcl2/TACI-Ig mice have bigger lymphoid follicles, due to a higher number of B-cells than wt controls. For Vav-Bcl2 mice it is already known that they are prone to develop follicular lymphoma, as displayed histologically and was (like the normal phenotype, the hypoplasia and the lymphoid hyperplasia) diagnosed by a pathologist.^2,6^ The images in Fig. 2(A)–(C) represent chemical maps. Fig. 2(A) depicts chemical maps generated by integrating the area under band absorption at 1080 cm^−1^, which is an indicator of the >PO_2_^−^ groups of nucleic acids and phospholipids. A symmetric phosphate as a distinguishing spectral marker was first proposed as a feature of stem cells by Walsh MJ *et al.*;^77^ this group later presented a distribution of this spectral marker associated with cancer stem cells.^78^ Fig. 2(B) depicts a chemical map generated by integrating the area under band absorption at 1155 cm^−1^, which is commonly attributed to carbohydrates. The result correlates well with the morphology of the red and white pulp, indicating that the B-lymphocytes within the lymphoid follicles produce a high amount of carbohydrates. The chemical map of the absorption at 1740 cm^−1^ is attributed to *ν*_C=O_ esters, phospholipids as well as carbohydrates (Fig. 2(C)). It is apparent that the signal intensities at 1740 cm^−1^ are higher in the hyperplastic and hypoplastic samples, compared to the lymphoma and normal samples. This is possible due to the fact that in the hyperplastic tissue, cells produce more *ν*_C=O_ esters, phospholipids as well as carbohydrates, compared to normal. Lymphoma cells are neoplastic and express, for instance, other or higher levels of certain cytokines, leading to alterations in the micro-environment and might influence the effectiveness of chemotherapy and subsequently the outcome.^79^

Results from the before mentioned individual tissue samples by HCA clustering, KMC clustering and fuzzy C-means clustering are illustrated in Fig. 3. The output of the data analyses illustrates the ability of spectroscopic imaging to reflect the tissue histology of samples. Tissue sections were measured with a nominal lateral resolution of 2.65 μm × 2.65 μm per pixel for each spot. The imaging results demonstrate that it is possible to acquire MIR images at a high resolution and that the results correspond to the tissue structures seen in the samples. Therefore, it is possible to correlate molecular signals detected by the used method with the histological features exhibited at a high resolution. MIAs were only implemented on individual MIR imaging data. To directly compare the spectra of defined ROIs of all different phenotypes, single spectral and principal component analyses (PCA) were performed.

Fig. 4(A) displays typical MIR transmission spectra of randomly selected regions obtained from the spleen tissue in the spectral region from 3900 cm^−1^ to 850 cm^−1^. Most prominent components are the amide A (N–H stretching vibrations of proteins, ~3300 cm^−1^), the C–H stretching vibrations (lipids, cholesterols and esters, ~3010 cm^−1^), the amide I-band (proteins, ~1620–1695 cm^−1^), the amide II-band (proteins, ~1550 cm^−1^), >CH_2_ deformation vibrations (~1468 cm^−1^) and the P=O symmetric stretching vibrations of the >PO^2−^ groups (phospholipids, nucleic acids ~1250–1220 cm^−1^). A direct comparison of the MIR spectra displayed no essential differences.

For further spectral analysis, principal component analyses (PCA) were applied to directly compare all different phenotypes with a statistical approach by using spectra of selected ROIs of the white pulp. PCA were performed to fully characterize the range of spectral variations. With PCA the dimensionality of MIR microscopy imaging spectra is reduced, while as much information as possible is retained. The scores of the first principal components are used to generate meaningful plots without a detailed understanding of the underlying sample biochemistry. For the PCA analysis across four different phenotype samples, 30 spectra were chosen. The results of spectral analyses with PCA are illustrated in Fig. 4(B) and (C).

The score plot of the first and the second principal component is based on 30 spectra of one specimen. For deploying PCA models, transmission spectra were converted to log(1/*R*). Additional pre-treatments for MIR spectra such as baseline offset and area normalization were utilized. The following wavenumber regions were tested for the PCA models: 3650 cm^−1^ to 850 cm^−1^, 3650 cm^−1^ to 3050 cm^−1^, 3000 cm^−1^ to 2800 cm^−1^, 1740 cm^−1^ to 1550 cm^−1^ and 1750 cm^−1^ to 850 cm^−1^. However, the PCA models indicate that most of the descriptive information can be found in the region from 1740 cm^−1^ to 1550 cm^−1^.

The score plots in Fig. 4(B) and (C) display a 2-D and 3-D visualization of spectral clusters for the principal component 1 explaining 99% of the total variance and can separate the different phenotype samples. This statistical strategy allows an easy feature extraction of several data sets and displays a distinct clustering according to the spectra obtained from four individual mouse genotypes with different phenotypes. However, it could not be determined to which extent this variation is caused due to qualitative and/or quantitative alterations.

## 4. Conclusion

MIR microscopy imaging methods have been widely used to characterize tissue samples; hence the benefits of this imaging method are obvious. It is ideally suited for sensitive detection of changes in the chemistry/biochemistry of tissues and is optimal for the establishment of a rapid, non-subjective, and cost-effective tool for diagnosis of cancer.

In the present study, MIR microscopy imaging and multivariate image analyses (MIAs) were used to gain deeper insight into the variations between different mouse phenotypes, particularly follicular hyperplasia caused by reactive lymphadenopathy and cancer (follicular lymphoma).

We were able to demonstrate, that with the mentioned sample preparation, measurement settings, and data analyses strategies, it is possible to get excellent MIR microscopy imaging results. Univariate MIR imaging results clarify that defined substance classes such as nucleic acids, phospholipids, carbohydrates, and esters could be imaged semi-quantitatively in different tissue types. Thus, MIR imaging could provide information about the molecular structure of the tissue under investigation. Specific correlations with histological traits and discrete chemical compounds, however, cannot be gauged with this form of processing.

Correlation of MIAs with morphological tissue features obtained by HE staining shows that many characteristics of the tissue can be visualized in the color cluster images. The different MIAs dramatically increase the information content of the IR data-sets and provide additional proof that tissue changes can be characterized by MIR microscopy imaging.

The best correlation between histopathology and spectral images was observed by HCA analyses. It is an unsupervised computational method in the sense, that neither the reference data, nor any starting conditions are required. By HCA analyses the number of clusters reproducing the best discrimination is selected. This could be achieved by terminating the calculations at a level, where the actual morphology is reproduced. Therefore, in terms of tissue structure differentiation, HCA clustering proved to be the best, but also the most calculation intensive image method, compared to KMC and FCM clustering.

With the help of principal component analyses (PCA) models, we were able to separate various genotypes in one statistical approach and, ultimately, differentiate between cancer, hyper- or hypoplastic and normal tissue.

## Abbreviations

BAFF: B-cell activating factor of the (TNF) family
Bcl2: B-cell lymphoma 2
CAML: Calcium-modulator and cyclophilin ligand
CCD: Charge coupled device
FCM: Fuzzy C-means
FDCs: Follicular dendritic cells
FTIR: Fourier transform infrared
FPA: Focal plane array
HE: Hematoxylin eosin
HCA: Hierarchical cluster analysis
IR: Infrared
KMC: K-means clustering
MIR: Mid infrared
MIAs: Multivariate imaging analysis
MCT: Mercury cadmium telluride
PBS: Phosphate-buffered saline
PCA: Principal component analyses
ROIs: Regions of interest
TACI: Transmembrane activator and CAML interactor
Ig: Immunoglobulin
TNF: Tumor necrosis factor
wt: Wild type
